# Are gait variability and stability measures influenced by directional changes?

**DOI:** 10.1186/1475-925X-13-56

**Published:** 2014-05-05

**Authors:** Federico Riva, Eleni Grimpampi, Claudia Mazzà, Rita Stagni

**Affiliations:** 1 DEI - Department of Electrical, Electronic, and Information Engineering ‘Guglielmo Marconi’, University of Bologna, Bologna, Italy; 2 Interuniversity Centre Of Bioengineering Of The Human Neuromusculoskeletal System, University of Rome “Foro Italico”, Rome, Italy; 3 Health Sciences and Technologies – Interdepartmental Center for Industrial Research (HST – ICIR), University of Bologna, Bologna, Italy; 4 INSIGNEO Institute for In Silico Medicine, The University of Sheffield, Sheffield, UK; 5 Department of Mechanical Engineering, The University of Sheffield, Sheffield, UK

**Keywords:** Gait, Directional change, Stability, Variability, Accelerometers

## Abstract

**Background:**

Many gait variability and stability measures have been proposed in the literature, with the aim to quantify gait impairment, degree of neuro-motor control and balance disorders in healthy and pathological subjects. These measures are often obtained from lower trunk acceleration data, typically acquired during rectilinear gait, but relevant experimental protocols and data processing techniques lack in standardization. Since directional changes represent an essential aspect of gait, the assessment of their influence on such measures is essential for standardization. In addition, their investigation is needed to evaluate the applicability of these measures in laboratory trials and in daily life activity analysis. A further methodological aspect to be standardized concerns the assessment of the sampling frequency, which could affect stability measures. The aim of the present study was hence to assess if gait variability and stability measures are affected by directional changes, and to evaluate the influence of sampling frequency of trunk acceleration data on the results.

**Methods:**

Fifty-one healthy young adults performed a 6-minute walk test along a 30 m straight pathway, turning by 180 deg at each end of the pathway. Nine variability and stability measures (Standard deviation, Coefficient of variation, Poincaré plots, maximum Floquet multipliers, short-term Lyapunov exponents, Recurrence quantification analysis, Multiscale entropy, Harmonic ratio and Index of harmonicity) were calculated on stride duration and trunk acceleration data (acquired at 100 Hz and 200 Hz) coming from straight walking windows and from windows including both straight walking and the directional change.

**Results:**

Harmonic ratio was the only measure that resulted to be affected by directional changes and sampling frequency, decreasing with the presence of a directional change task. HR was affected in the AP and V directions for the 200 Hz, but only in AP direction for the 100 Hz group.

**Conclusion:**

Multiscale entropy, short term Lyapunov exponents and Recurrence quantification analysis were generally not affected by directional changes nor by sampling frequency, and could contribute to the definition of a fall risk index in free-walking conditions.

## Introduction

The quantification of gait variability and stability is performed in the literature by means of several measures [1-7], often based on lower trunk acceleration data. These measures aim to quantify gait impairment, degree of neuro-motor control and balance disorders in different subjects. However, methodological standardization is lacking for their wider exploitation.

Despite often been ignored in gait analysis, directional changes represent an essential aspect of gait. Directional changes can occur both in controlled clinical/laboratory trials (e.g. Timed Up and Go test [8-10], 6-minute walk test [11]) and in daily activities, since 20-50% of steps performed during daily activities are reported to be turns [12,13], ranging from a few degrees to a full 180 deg turn. The influence of directional changes on variability and stability measures must be evaluated, and validity of such measures in presence of turns during walking is hence investigated. To this aim, we choose to compare straight walking trials with trials in which a directional change was present. Having to choose the sharpness of the directional change for our experimental analysis, our research hypothesis was that hazardous turns (180 degrees) would have a higher influence on variability and stability measures compared to minor turns; the eventual absence of such a major turn influence would suggest that the presence of directional changes of smaller entity would have little to no effects on variability and stability measures. This is particularly important in overground walking tasks, when completely straight walking direction is difficult to assure. According to recent research, few steps are not sufficient for the reliable quantification of stability indexes and long straight paths are often not available [14].

It has been proven that some measures can be influenced by the acquisition sampling frequency [12,15], and that sampling frequency should hence be taken into account when trying to obtain a methodological standard for the clinical use of these measures. These studies, however, are solely based on the analysis of joint angles of lower limbs. In the search for a methodological characterization and a broader applicability, the influence of sampling frequency on variability and stability measures obtained from trunk accelerations needs to be investigated as well.

The aim of this study was to preliminarily assess the influence of directional changes and sampling frequency on gait variability and stability measures calculated on lower trunk acceleration data, in order to establish if these experimental conditions can have a disruptive impact on results. A sample of healthy young subjects walking in straight walking condition and in presence of directional changes was investigated.

## Methods

Fifty-one healthy young adults (23 ± 3 years, 172 ± 11 cm, 68 ± 14 kg) volunteered for this study. Participants were chosen among students involved in sports activities promoted by the University of Rome “Foro Italico”. All subjects were physically active and self-reported no musculoskeletal or neurological disorders that could affect their performance and/or behaviour. The Review Board Committee of the authors’ institution approved this study, and informed consent was obtained from the participants.

Participants performed a 6-minute walk test [11]. In detail, they were asked to walk back and forth for 6 minutes along a 30 m straight pathway, turning by 180 deg at each end of the pathway, and to cover the maximum possible distance during the 6 minutes and, thus, walking as fast as possible. Average gait speed was 1.37 ± 0.15 m/s, estimated using the method proposed by Zijlstra and Hof [16]. The fast speed and the 180 deg turn were considered in order to test the limit condition in terms of gait instability, representing a very sharp and potentially hazardous directional change.

An inertial measurement unit (FreeSense, Sensorize s.r.l) was fixed to the lower trunk of the subjects at the level of the fifth lumbar vertebra through an elastic neoprene belt; the sensor local axes were aligned to the anterior-posterior, medio-lateral and longitudinal trunk axes, respectively. To ensure standardization, sensor placements were performed by the same operator based on the identification of anatomical landmarks on the subjects. Local axes of the sensor were aligned to the trunk in order to obtain a vertical axis pointing up and parallel to the longitudinal axis of the trunk, an anterior-posterior axis pointing forward and perpendicular to the trunk and a medio-lateral axis pointing left.

Data from the gyroscopes was used to identify turns and straight walking intervals, but only acceleration data were considered for the analysis.

Subjects were randomly divided into two matched groups of n = 25 and n = 26 subjects. One trial per subject was acquired for the first group with a sampling frequency equal to 100 Hz and one trial per subject was acquired at 200 Hz for the second group. A third set of data was then obtained from the second group, down-sampling acceleration signals from 200 Hz to 100 Hz, and added to the 100 Hz group. Experimental data were analyzed without filtering, in order to avoid the complications associated with the application of linear filtering to nonlinear signals [17] and to allow comparison with other studies [18]. Foot strikes were detected from the vertical acceleration using the algorithm proposed by McCamley et al. [19]. Walking data were divided into two separate portions (about 20 strides each); portions in which the subject walked in a straight line were labeled SW, while portions in which the subject underwent a directional change during the walk were labeled DCW. The number of strides was chosen as the maximum number of strides reachable by the subjects in completely straight walking conditions. Stride durations were obtained as the time intervals between two consecutive heel-strikes of the same foot.

Eight variability and stability measures were calculated. The choice of the measures to include in the analysis was made based on their popularity in research and clinical settings and with the aim to cover a wide range of acceleration signal features (stability, recurrence, complexity, smoothness and harmonicity). Three temporal variability measures were applied to stride duration: Standard deviation (SD) [1], Coefficient of variation (CV) [1] and Poincaré plots (PSD1, PSD2) [2]. Stride durations were obtained as the time intervals between two consecutive heel-strikes of the same foot. Five stability measures were calculated on trunk acceleration components in the vertical (V), medio-lateral (ML) and anterior-posterior (AP) directions: short-term Lyapunov exponents (sLE) [3], Recurrence quantification analysis (RQA) [4], Multiscale entropy (MSE) [5], Harmonic ratio (HR) [6] and Index of harmonicity (IH) [7].

Additional information about variability and stability measures is illustrated in the Appendix, together with details about implementation parameters.

Inconsistency of variance (IV) [1], Nonstationary index (NI) [1], long-term Lyapunov exponents (lLE) [3] and RQA (max, diverg) [4] were also considered, but the 20 stride sample was deemed not sufficient to draw accurate conclusions, because these indexes were assessed to have an intrinsic variability > 50% when calculated on 20 strides [20]. Since gait data have been proved to be both nonlinear as well as non-stationary [21], all of these stability measures account for non-stationarity. Details on the measures are illustrated in Table 1.

**Table 1:** **Details on intrinsic variability of measures for 20 strides and reference *imr *** **calculated on long overground walks performed by a sample of healthy young subjects and use for analysis **[20]

	**Measures**	**Variability for 20 strides**		***imr***
Temporal variability measures	Standard deviation (SD)		20-30%	0,10
	Coefficient of variation (CV)		30-40%	0,10
	Inconsistency of variance (IV)		> 50%	0,20
	Nonstationary index (NI)		> 50%	0,24
	Poincaré plots	PSD1	20-30%	0,07
		PSD2	40-50%	0,14
Stability measures	Short-term Lyapunov exponents (sLE)	tot	20-30%	0,26
		AP	10-20%	0,20
		ML	10-20%	0,18
		V	10-20%	0,20
	Long-term Lyapunov exponents (lLE)	tot	> 50%	0,29
		AP	> 50%	0,33
		ML	> 50%	0,30
		V	> 50%	0,22
	Recurrence quantification analysis (RQA)	rr AP	< 10%	0,01
		rr ML	< 10%	0,01
		rr V	< 10%	0,02
		det AP	10-20%	0,02
		det ML	10-20%	0,03
		det V	10-20%	0,01
		avg AP	10-20%	0,03
		avg ML	10-20%	0,02
		avg V	10-20%	0,03
		max AP	> 50%	0,17
		max ML	> 50%	0,26
		max V	> 50%	0,42
		diverg AP	> 50%	0,27
		diverg ML	> 50%	0,23
		diverg V	> 50%	0,54
	Multiscale entropy (MSE)	AP τ = 1	10-20%	0,02
		AP τ = 2	10-20%	0,03
		AP τ = 3	10-20%	0,03
		AP τ = 4	10-20%	0,03
		AP τ = 5	10-20%	0,03
		AP τ = 6	10-20%	0,03
		ML τ = 1	10-20%	0,02
		ML τ = 2	10-20%	0,03
		ML τ = 3	10-20%	0,03
		ML τ = 4	10-20%	0,03
		ML τ = 5	10-20%	0,03
		ML τ = 6	10-20%	0,03
		V τ = 1	10-20%	0,03
		V τ = 2	10-20%	0,03
		V τ = 3	10-20%	0,03
		V τ = 4	10-20%	0,04
		V τ = 5	10-20%	0,03
		V τ = 6	10-20%	0,04
	Harmonic ratio (HR)	AP	20-30%	0,07
		ML	20-30%	0,06
		V	20-30%	0,07
	Index of harmonicity (IH)	AP	40-50%	0,20
		ML	30-40%	0,17
		V	30-40%	0,21

In order to assess the influence of directional changes on the measures, statistical differences in results between SW and DCW conditions were investigated. Z-scores between the two conditions were obtained for each subject and each measure calculated separately on acceleration components (AP, ML and V) for the two sampling groups (100 Hz and 200 Hz). As a measure of variance, previously found reference values of interquartile range/median ratio (*imr*) calculated on a long overground walk performed by young subjects were used [20]. These values are reported in Table 1. Bonferroni-corrected p-values for each measure at each sampling condition were then calculated based on the z-scores. The capability of the measures to discriminate between SW and DCW conditions (p < 0.05) in the majority of the subjects (>20 for 200 Hz group, > 40 for 100 Hz group) was assessed. The increasing or decreasing effect of directional changes was also assessed, based on the sign of the mean value of the difference between measures obtained in SW and DCW conditions.

An additional analysis was conducted performing a two tails paired t-test in order to compare mean values of measures in the SW and DCW conditions. Effect size (Cohen’s *d*) and Power of the study have also been calculated.

## Results and discussion

Only HR was affected by directional changes, both at 200 Hz and at 100 Hz. HR decreased when a directional change was present in the task. HR was affected in the AP and V directions for the 200 Hz, but only in AP direction for the 100 Hz group.

Other measures (SD, CV, PSD1, PSD2, MSE, RQA, maxFM and sLE) were found to be affected neither by directional changes nor by sampling frequency during walking. Given the number of measures analyzed, we are only reporting results in the text, since a table including p-values for all the measures would carry little information compared to its size.

Student’s t-test showed no significant differences (p-value > 0.05) between the two groups. Effect sizes were generally medium for both 200 Hz and 100 Hz conditions, whereas Power was generally high (around 0.8) for 200 Hz condition and a little lower for 100 Hz condition.

Mean values of variability/stability measures among subjects in the different conditions are shown, together with standard deviations and t-test results, in Tables 2 and 3, respectively.

**Table 2:** Mean values and standard deviations of variability/stability measures among subjects (n = 26) calculated between SW and DCW conditions at 200 Hz

	**SW (200 Hz)**		**DCW (200 Hz)**				
	**Mean**	**std**	**Mean**	**std**	**p-value**	**Cohen’s *d***	**Power**
MSE V *τ* = 1	0,42	0,06	0,43	0,05	0,74	0,18	0,76
MSE V *τ* = 2	0,59	0,09	0,61	0,08	0,80	0,23	0,67
MSE V *τ* = 3	0,73	0,13	0,76	0,13	0,79	0,23	0,68
MSE V *τ* = 4	0,85	0,16	0,88	0,16	0,75	0,19	0,75
MSE V *τ* = 5	0,95	0,19	0,98	0,17	0,72	0,17	0,79
MSE V *τ* = 6	1,03	0,2	1,07	0,18	0,77	0,21	0,72
MSE ML *τ* = 1	0,49	0,09	0,49	0,09	0,50	0,00	0,95
MSE ML *τ* = 2	0,69	0,11	0,69	0,11	0,50	0,00	0,95
MSE ML *τ* = 3	0,89	0,15	0,9	0,15	0,59	0,07	0,90
MSE ML *τ* = 4	1,08	0,18	1,1	0,19	0,65	0,11	0,86
MSE ML *τ* = 5	1,26	0,21	1,27	0,22	0,57	0,05	0,92
MSE ML *τ* = 6	1,4	0,21	1,42	0,22	0,63	0,09	0,88
MSE AP *τ* = 1	0,25	0,06	0,26	0,05	0,74	0,18	0,76
MSE AP *τ* = 2	0,41	0,08	0,42	0,08	0,67	0,13	0,84
MSE AP *τ* = 3	0,53	0,11	0,55	0,1	0,75	0,19	0,75
MSE AP *τ* = 4	0,63	0,13	0,66	0,12	0,80	0,24	0,66
MSE AP *τ* = 5	0,71	0,15	0,75	0,13	0,84	0,28	0,58
MSE AP *τ* = 6	0,78	0,15	0,81	0,14	0,77	0,21	0,72
RQA V (rr)	15,25	1,86	14,62	1,88	0,88	0,34	1,00
RQA V (det)	88,1	3,07	88,84	2,86	0,81	0,25	0,65
RQA V (avg)	19,81	3,91	18,37	3,26	0,92	0,40	1,00
RQA ML (rr)	9,86	1,42	9,46	1,25	0,85	0,30	1,00
RQA ML (det)	75,71	8,22	75,94	7,71	0,54	0,03	0,93
RQA ML (avg)	9,43	1,76	9,18	1,59	0,70	0,15	0,99
RQA AP (rr)	18,2	1,4	17,9	1,51	0,77	0,21	1,00
RQA AP (det)	89,16	2,15	89,87	2,1	0,88	0,33	0,48
RQA AP (avg)	14,04	2,13	14,05	2,19	0,51	0,00	0,95
HR V	2,12	1,37	2,02	1,16	0,61	0,08	0,98
HR ML	0,82	0,4	0,92	0,41	0,81	0,25	0,65
HR AP	2,33	1,57	1,87	1,02	0,89	0,35	1,00
IH V	0,06	0,05	0,04	0,02	0,96	0,53	1,00
IH ML	0,22	0,1	0,19	0,11	0,84	0,29	1,00
IH AP	0,07	0,06	0,06	0,04	0,76	0,20	1,00
PSD1	0,03	0,02	0,05	0,04	0,98	0,63	0,06
PSD2	0,02	0,01	0,04	0,06	0,95	0,46	0,23
SD	0,03	0,01	0,05	0,05	0,97	0,55	0,12
CV	3,64	1,74	5,81	7,56	0,92	0,40	0,35
sLE tot	0,7	0,16	0,74	0,16	0,81	0,25	0,64
sLE AP	1,22	0,23	1,34	0,19	0,97	0,57	0,10
sLE ML	1,54	0,33	1,62	0,3	0,82	0,25	0,64
sLE V	1,52	0,24	1,62	0,32	0,89	0,35	0,44

Student test’s p-values, Cohen’s *d* and Power of the study are also shown.

**Table 3:** Mean values and standard deviations of variability/stability measures among subjects (n = 51) calculated between SW and DCW conditions at 100 Hz

	**SW (100 Hz)**		**DCW (100 Hz)**				
	**Mean**	**std**	**Mean**	**Std**	**p-value**	**Cohen’s *d***	**Power**
MSE V *τ* = 1	0,56	0,09	0,59	0,08	0,96	0,35	0,19
MSE V *τ* = 2	0,83	0,15	0,87	0,14	0,91	0,28	0,37
MSE V *τ* = 3	1,01	0,19	1,06	0,18	0,91	0,27	0,39
MSE V *τ* = 4	1,1	0,2	1,16	0,2	0,93	0,30	0,31
MSE V *τ* = 5	1,12	0,2	1,2	0,21	0,97	0,39	0,13
MSE V *τ* = 6	1,17	0,21	1,21	0,21	0,83	0,19	0,61
MSE ML *τ* = 1	0,67	0,09	0,68	0,09	0,71	0,11	0,80
MSE ML *τ* = 2	1,08	0,16	1,09	0,16	0,62	0,06	0,88
MSE ML *τ* = 3	1,38	0,2	1,41	0,19	0,78	0,15	0,71
MSE ML *τ* = 4	1,59	0,21	1,61	0,2	0,69	0,10	0,83
MSE ML *τ* = 5	1,65	0,19	1,72	0,19	0,97	0,37	0,16
MSE ML *τ* = 6	1,69	0,21	1,75	0,18	0,94	0,31	0,29
MSE AP *τ* = 1	0,41	0,09	0,42	0,08	0,72	0,12	0,79
MSE AP *τ* = 2	0,64	0,13	0,66	0,12	0,79	0,16	0,69
MSE AP *τ* = 3	0,8	0,16	0,84	0,15	0,90	0,26	0,42
MSE AP *τ* = 4	0,91	0,16	0,94	0,16	0,83	0,19	0,62
MSE AP *τ* = 5	0,96	0,13	1	0,16	0,91	0,27	0,38
MSE AP *τ* = 6	0,98	0,16	1,01	0,15	0,83	0,19	0,60
RQA V (rr)	15,44	2,39	14,67	2,22	0,95	0,33	1,00
RQA V (det)	76,08	6,98	76,99	7,5	0,74	0,13	0,77
RQA V (avg)	14,21	3,56	13,17	3,07	0,94	0,31	1,00
RQA ML (rr)	10,2	2,14	9,69	1,78	0,90	0,26	1,00
RQA ML (det)	46,71	12,51	44,61	11,85	0,81	0,17	1,00
RQA ML (avg)	7,6	1,15	7,21	0,77	0,98	0,40	1,00
RQA AP (rr)	18,14	1,54	17,83	1,45	0,85	0,21	1,00
RQA AP (det)	74,05	8,31	75,68	7,73	0,84	0,20	0,58
RQA AP (avg)	8,9	1,34	8,83	1,26	0,61	0,05	0,98
HR V	2,4	1,61	2,38	1,2	0,53	0,01	0,96
HR ML	0,79	0,4	0,83	0,42	0,69	0,10	0,83
HR AP	2,37	1,49	2,12	1,11	0,83	0,19	1,00
IH V	0,05	0,04	0,04	0,02	0,94	0,32	1,00
IH ML	0,21	0,11	0,19	0,1	0,83	0,19	1,00
IH AP	0,08	0,06	0,06	0,03	0,98	0,42	1,00
PSD1	0,02	0,02	0,04	0,03	1,00	0,78	0,00
PSD2	0,02	0,01	0,04	0,04	1,00	0,69	0,00
SD	0,03	0,02	0,04	0,04	0,94	0,32	0,27
CV	2,9	2,03	5,12	5,73	0,99	0,52	0,02
sLE tot	0,6	0,17	0,66	0,17	0,96	0,35	0,19
sLE AP	0,73	0,18	0,8	0,17	0,98	0,40	0,11
sLE ML	0,78	0,21	0,83	0,16	0,91	0,27	0,39
sLE V	0,79	0,2	0,86	0,18	0,97	0,37	0,16

Student test’s p-values, Cohen’s *d* and Power of the study are also shown.

Turning is a fundamental aspect of everyday walking, and it has been identified as more challenging than straight-line walking for old adults and gait-impaired subjects [8,9]. Moreover, some reports have shown that turns can be predictive of dysfunction in older adults with and without neurological disorders [22]. When wanting to analyze long overground walking data for gait variability and stability analysis purposes, turns may have to be taken into account, since long straight paths are often not available. In the methodological standardization of gait variability and stability measures based on lower trunk acceleration, we addressed the not previously investigated influence of directional changes on such measures.

The measurement of the gait smoothness and rhythmicity, i.e. the HR [6], was found to be affected by directional change when calculated on the AP and V acceleration components, but not on the ML component. HR provides an indication of the smoothness and rhythm of acceleration patterns, based on the premise that the unit of measurement from a continuous walking trial is a stride [6]; it is hence perhaps not surprising that a sharp turn may introduce out of phase harmonics, heavily influencing the measure. The effect was observed in AP and V directions, but not in ML direction; it is likely that the alternation of right/left steps is maintained even during a turn, allowing the trunk to keep its medio-lateral oscillating pattern almost unvaried.

The sampling frequency affected the measures, but not for all the acceleration directions. At 100 Hz, only HR in the AP direction was found to be affected by directional change, while at 200 Hz AP and V directions were affected. This is likely caused by the loss of information induced by the lower sampling frequency.

IH, maxFM, sLE, MSE and RQA were affected neither by directional changes nor by sampling frequency; harmonicity, orbital/local stability, entropy and recurrence of trunk acceleration signals were comparable between straight line gait and gait with directional changes. Measures aimed at quantifying such characteristics are hence exploitable also in settings in which completely straight line gait is not achievable.

Also the variability measures based on stride duration (SD, CV, PSD1, PSD2) were unaffected by directional changes and sampling frequency. It is likely that the variations in stride durations were small during the 180 deg turn, hence not significantly influencing measures based on its variability.

The direct comparison of the two distributions (SW and DCW) via t-test didn’t highlight any significant difference between the mean value of measures obtained in the two conditions. The average detectable effect size was 0.24 for the 200 Hz condition and 0.27 for the 100 Hz condition.

Even though the subjects spent only a small amount of time turning in the DCW condition, acceleration signals underwent modifications during the directional change. The influence of such modification on variability/stability measures was found to be negligible for almost all the measures considered. It cannot be excluded that changing the ratio between time spent walking and time spent turning (i.e. analyzing less strides or gait on a winding path) would have led to different results, and maybe to a higher number of measures sensitive to turns. However, we believe that such results would be less meaningful for clinical/research application of variability and stability measures. Experimental conditions often imply quite long straight-line walking data with a few directional changes in it, in order to cope with the limited available space of a laboratory environment; in order to obtain realistic and exploitable results, a worst-case scenario based on this usual experimental setting had to be recreated.

## Conclusion

The overall absence of a major influence of directional changes on variability and stability measures suggests that such measures could be also calculated in presence of turns without losing validity; since turns do not affect most variability/stability measure, any change observed in the measures is likely to be caused by an actual change in the locomotor stability of the subject. This is particularly relevant in the analysis of overground walking, in which perfectly straight walking conditions are harder to obtain, allowing researchers to exclude the presence of directional changes in the task as a possible source of error. Sampling frequency seemed also to have no influence on variability and stability measures, except for HR.

In conclusion, HR was the only measure affected by directional changes and sampling frequency, and hence it could be unreliable in overground free walking conditions. In particular, MSE, sLE and RQA were not affected by the presence of turns during the walk; having also recently proved to be related to fall history in treadmill walking tests [23,24], such measures could contribute to the definition of a fall risk index in free-walking conditions. Further research is needed to assess the capability of these measures to identify fall-prone subjects in an over-ground walking task.

## Appendix

### Variability measures

Standard Deviation (SD) has simply been calculated as the standard deviation of stride duration.

Coefficient of Variation (CV) has been calculated as the variability of stride duration normalized to the mean stride duration value (CV = 100 × SD / mean) [25].

Inconsistency of Variance (IV) and Nonstationary Index (NI) quantify the temporal “structure” of the time series (independent of the overall variance). Each time series was first normalized with respect to its mean and SD, and then divided into blocks of five strides each. In each segment, the local average and the local SD were computed.

Stride duration data plots between successive gait cycles, known as Poincaré plots, show the variability of stride duration data. Statistically, the plots display the correlation between consecutive stride durations data in a graphical manner. PSD1 and PSD2 represent, respectively, width and length of the long and short axis of the elliptical plots, and hence the short-term and long-term variability of stride duration [2].

### Stability measures

Short-term Lyapunov Exponents (sLE) quantify local dynamic stability of a system and are used for systems that do not necessarily exhibit a discernable periodic structure [3]. Recurrence Quantification Analysis (RQA) provides a characterization of a variety of features of a given time series, including a quantification of deterministic structure and non-stationarity [4], based on the construction of recurrence plots [26].

These measures imply the reconstruction of the state space of the system; in this study, four different state spaces were constructed: one 3-dimensional state space composed by the V, ML and AP accelerations and three (one per direction) 5-dimensional state spaces composed by delay-embedding of each acceleration component (delay = 10 samples) [15,27,28]. Several measures were then extracted from RQA, namely recurrence rate (rr), determinism (det) and averaged diagonal line length (avg), using a radius of 40%.

Multiscale Entropy (MSE) quantifies the complexity or irregularity of a time series [29]. MSE has been obtained calculating sample entropy (consecutive data points *m* = 2, distance *r* = 0.2 [30]) on six consecutively more coarse-grained (scale factor *τ* = 1, …, 6) time series.

Harmonic Ratio (HR) provides information on how smoothly subjects control their trunk during walking and gives an indication of whole body balance and coordination [6,31].

Similarly to HR, Index of Harmonicity (IH) quantifies the contribution of the stride frequency to the signal power relative to higher harmonics Y.

## Competing interests

The authors report no conflict of interest relevant to the subject of this article.

## Authors’ contribution

FR participated in the design of the study, carried out the data elaboration and drafted the manuscript. EG participated in the data acquisition and helped to draft the manuscript. CM participated in the design of the study, carried out the data acquisition and helped to draft the manuscript. RS conceived of the study, participated in its design and coordination and helped to draft the manuscript. All authors read and approved the final manuscript.
